# Toward Successful Implementation of Artificial Intelligence in Health Care Practice: Protocol for a Research Program

**DOI:** 10.2196/34920

**Published:** 2022-03-09

**Authors:** Petra Svedberg, Julie Reed, Per Nilsen, James Barlow, Carl Macrae, Jens Nygren

**Affiliations:** 1 School of Health and Welfare Halmstad University Halmstad Sweden; 2 Department of Health, Medicine and Caring Sciences Linköping University Linköping Sweden; 3 Centre for Health Economics and Policy Innovation Imperial College Business School London United Kingdom; 4 Centre for Health Innovation, Leadership and Learning Nottingham University Business School Nottingham United Kingdom

**Keywords:** process evaluation, complex intervention, implementation, knowledge exchange, health policy, organizational change, capacity building, qualitative methods, framework analysis

## Abstract

**Background:**

The uptake of artificial intelligence (AI) in health care is at an early stage. Recent studies have shown a lack of AI-specific implementation theories, models, or frameworks that could provide guidance for how to translate the potential of AI into daily health care practices. This protocol provides an outline for the first 5 years of a research program seeking to address this knowledge-practice gap through collaboration and co-design between researchers, health care professionals, patients, and industry stakeholders.

**Objective:**

The first part of the program focuses on two specific objectives. The first objective is to develop a theoretically informed framework for AI implementation in health care that can be applied to facilitate such implementation in routine health care practice. The second objective is to carry out empirical AI implementation studies, guided by the framework for AI implementation, and to generate learning for enhanced knowledge and operational insights to guide further refinement of the framework. The second part of the program addresses a third objective, which is to apply the developed framework in clinical practice in order to develop regional capacity to provide the practical resources, competencies, and organizational structure required for AI implementation; however, this objective is beyond the scope of this protocol.

**Methods:**

This research program will use a logic model to structure the development of a methodological framework for planning and evaluating implementation of AI systems in health care and to support capacity building for its use in practice. The logic model is divided into time-separated stages, with a focus on theory-driven and coproduced framework development. The activities are based on both knowledge development, using existing theory and literature reviews, and method development by means of co-design and empirical investigations. The activities will involve researchers, health care professionals, and other stakeholders to create a multi-perspective understanding.

**Results:**

The project started on July 1, 2021, with the Stage 1 activities, including model overview, literature reviews, stakeholder mapping, and impact cases; we will then proceed with Stage 2 activities. Stage 1 and 2 activities will continue until June 30, 2026.

**Conclusions:**

There is a need to advance theory and empirical evidence on the implementation requirements of AI systems in health care, as well as an opportunity to bring together insights from research on the development, introduction, and evaluation of AI systems and existing knowledge from implementation research literature. Therefore, with this research program, we intend to build an understanding, using both theoretical and empirical approaches, of how the implementation of AI systems should be approached in order to increase the likelihood of successful and widespread application in clinical practice.

**International Registered Report Identifier (IRRID):**

PRR1-10.2196/34920

## Introduction

### Background

In many high-income countries, policy makers, authorities, and care providers have great expectations that the uptake of information technology (IT) innovations in health care will contribute to improved efficiency and quality of health care as well as improved clinical and health outcomes [1,2]. Today, artificial intelligence (AI), as an IT innovation, holds significant promise for enhancing health care [3]. However, as for most other types of IT innovations, the uptake of AI in health care is still at an early stage [4]. Even though there are ample examples of successful implementation of innovations in health care practice, there are often considerable challenges to implement new technology in health care [5]. While implementation science has advanced our knowledge about barriers to implementing such innovations and provided guidance about what strategies can be used to overcome these barriers, this knowledge has not yet been applied for understanding or supporting the implementation of AI in health care [6].

Despite the extensive and still increasing interest in and research on AI in health care, it is evident that there is a huge knowledge gap on how to tackle implementation challenges and how to successfully plan to increase the likeliness of sustainable adoption in practice [7-9]. To date, research on AI in health care has mostly focused on algorithm development, proof-of-concept evaluations, and technical, legal, and ethical challenges [3,8,10-12]. The research is primarily published within the fields of computer science, engineering, and medical informatics [7] as well as the clinical specialties of oncology, neurology, radiology, and cardiology [3].

A variety of viewpoint articles, commentaries, and guidelines have been produced, discussing the potential of AI but also the challenges and aspects to consider in its development and introduction in health care [8,11,13,14]. However, such articles are mainly part of a scientific discourse built on an underdeveloped evidence base. This reduces their value as guidance of implementation initiatives in practice. The research literature, in the form of systematic reviews, that is relevant to implementation-related issues has mostly addressed aspects of regulation, privacy, and legal issues [15,16]; ethics [9,16,17]; clinical and patient outcomes [18-20]; and economic impact [21]. These studies point to the importance of undertaking more implementation research to study AI implementation in real-world clinical settings.

A few empirical studies with robust methodology, such as randomized controlled studies, have investigated the effects of implementation of AI technology in practice [20], but there are no AI-specific implementation theories, models, or frameworks that could provide guidance for how to translate the potential of AI into daily health care practices [22]. Thus, there is currently a paucity of knowledge concerning several key issues, including barriers and facilitators to successful implementation of AI in health care; what strategies might be used to support AI implementation; how the use of AI might change existing clinical workflows, roles, and responsibilities; or how the infrastructure of management and governance should be constructed to be effective [23].

Many barriers to optimal implementation of new innovations in health care have been identified. Among these is the alignment of the innovation with the setting and contextual circumstances in which the innovation will be used, and the degree of adaptation to the needs and wants of the stakeholders that the innovation is intended to support. Such barriers often result in poor program fidelity and a lack of sustainability in change behavior at individual, organizational, and system levels from the innovation [24,25]. A general dilemma is how to involve both early adopters and the majority of health care professionals. While early adopters usually are fairly easy to engage and are motivated in the development and use of new innovations, the large group of health care professionals represents much greater variation with regard to their willingness to be involved and in motivation to integrate the use of innovations in their everyday practice [26]. There is also a general lack of strategic knowledge on how to ensure that innovations can be safely and effectively integrated into local infrastructures for routine use and embedded into clinical workflows [27].

In order to meet these challenges, implementation models, frameworks, and strategies are needed that contain multiple components embedded in the context of application [28,29]. In particular, coproduction has been stressed as a key factor for successful implementation of innovations in health care in order to develop effective strategies and to ensure that value is created [30]. This requires knowledge about both barriers and facilitators that influence an innovation’s use and implementation strategies that are designed to overcome the identified barriers and maximize the use of facilitating factors. Successful implementation usually requires an active change process aimed to achieve both organizational- and individual-level use of the intervention as designed. However, implementation is often a critical process between an organizational decision to adopt and support an innovation and the professional’s willingness and ability to use it in their daily work. In order for AI to be successfully introduced to change clinical practice, we need to understand current practices and the contexts in which those practices are conducted, as well as how AI would fit with or change those ongoing practices and processes. However, the experiences of the professionals and patients who use a particular AI application are often overlooked [12]. It is, therefore, important to understand how care is actually delivered, how data are currently used to inform health care, and how new technologies impact individual and organizational decision-making processes and alter the roles, responsibilities, and relationships that shape clinical work.

Given that health care practitioners have an ethical and legal duty of care to their patients and are responsible for clinical recommendations and decision-making, transparency regarding how clinical decisions are made—both with and without the use of AI—is important. This will necessitate the implementation of systems that encourage health care professionals to interact with AI in ways that augment their clinical decision-making without compromising their primary responsibilities and duties to patient care [31]; as well, implemented systems will need to ensure that quality and safety are appropriately governed and assured [32]. In order to achieve successful implementation, we need to close the gap between how work is imagined and what is actually taking place, and we need to build accurate, evidence-based, and shared understanding of what is really happening. Thus, more rigorous and empirical implementation studies of AI in health care, underpinning strong theory and real-world understanding, are urgently needed. 

### Objectives and Aims

This paper provides an outline for the first 5 years of a program lasting 8 years, using implementation and improvement frameworks and co-design between researchers, health care professionals, and stakeholders. There are two objectives for this initial period of the program and a third objective for the final period (not covered by this paper): 

1. To develop a theoretically informed framework for AI implementation in health care that can be applied to facilitate such implementation in routine health care practice. 
2. To carry out empirical AI implementation studies guided by the AI implementation framework, thus producing evidence on the value of the framework and generating learning for enhanced knowledge and operational insights to guide further refinement of the framework. 
3. To practically apply the developed framework in clinical practice to develop regional capacity to provide the practical resources, competencies, and organizational structure required for AI implementation.

## Methods

### Setting

This research program is part of a regional and national initiative to build infrastructure to support the implementation of AI into practice. Together, Halmstad University (HU) and Region Halland (RH) have appointed the implementation of AI in health care as a prioritized cooperation area for research and innovation with the aim to accomplish more information-driven care. Together with a broad cluster of business partners, HU and RH have established a research center for information-driven care called CAISR (Center for Applied Intelligent Systems Research) Health, with funding provided by the Knowledge Foundation (a Swedish funding agency). 

The CAISR Health research center builds on multidisciplinary collaboration between academics with expertise in data analytics, digital service, health economics, health care implementation, and health management. The center is unique in that it brings all these competencies together with partners in regional and municipal health care and industry in a joint undertaking to promote the use of information-driven care approaches in clinical practice. Emphasis has been placed on research to achieve a broad and deep understanding of how AI can be successfully implemented in health care. AI, and its implementation, in this context is broadly defined as the use, primarily, of machine learning but also other sophisticated computational techniques on health care data to support and improve clinical workflows, processes, and systems to improve quality and optimize resource use. This includes several layers within the sociotechnical ecosystem around the technology, dealing with (1) generating, cleaning, and labeling data; (2) developing models and verifying, assuring, and auditing AI tools and algorithms; (3) incorporating AI outputs into clinical decisions and resource allocation; and (4) the shaping of new organizational structures, roles, and practices. This implies that the implementation of AI extends beyond any specific intelligent technology and encompasses the whole sociotechnical system that surrounds and supports a particular technology. Given this, the focus of implementation is this broader AI system; therefore, AI is hereafter referred to as AI systems.

Realizing the ambition of successfully implementing AI systems in health care thus requires more than merely technological development. It also depends on knowledge about social, cultural, organizational, and implementation challenges; how information-driven care can be supported by AI systems; and what value can be created from different perspectives throughout the health care system.

Several major investments have been made in infrastructure and various forms of collaboration to achieve the ambition of high-quality research on and development of information-driven care.

One major investment was a research group for health care improvement that was developed at HU, with national and international collaborations with academic partners. The unit is interdisciplinary and combines applied and theoretical approaches, using both qualitative and quantitative methods, and is built up through extensive collaboration with users, regions, municipalities, and industry. The research focuses on questions about how health innovations, in the form of interventions supported by digital services and health data analysis, can be developed, implemented, and evaluated to provide health care organizations with knowledge and support to achieve high-quality care and improved health outcomes for particular groups. 

Another major investment was a national effort that was initiated to establish an innovation environment for information-driven care to improve Swedish health care. The initiative, which is funded by the Swedish innovation agency Vinnova, aims to develop health care to become more information driven, individualized, and scalable through implementation and use of AI systems. This is done in close collaboration with public, private, and academic parties. Both HU and RH have key roles as innovators, and the environment involves both the Swedish Association of Local Authorities and Regions and AI Sweden. 

An additional major investment was an innovation arena, Leap for Life, for information-driven care that was established at HU with the purpose to act as a driving force for collaborations, innovations, and change within health care, from regional, national, and international perspectives. Leap for Life has its premises at HU and was built in partnership with RH and all the municipalities in Halland. 

The final major investment was a strategic health care analysis and research platform, Regional Healthcare Information Platform (RHIP), which was established to enable agile management and analysis of the clinical and administrative data of every consumer of regional health care; the platform has had public funding since 2009 [33]. The platform is a structured, filtered, and pseudoanonymized far-reaching subset of the different data warehouses of patient and administrative data collected from over 20 different regional IT systems and national registers. It is designed to facilitate rapid analysis for clinical and management research and evaluation purposes. The Center for Information-Driven Care (CIDD) is a core facility within RH with responsibility for analyzing, simulating, and following up the effects of changes to the health care system at the system level and combining assessments of quality and costs. CIDD’s mission is to perform system analyses, based on machine learning models; to be able to support change in and assure the quality of health care processes; to manage and develop the analytical platform and its methods; and to support research in the area. A research platform in the form of a Health Data Center (HDC), with a similar structure and content as RHIP, is located at HU and was established to enable the use of the content in RHIP for research and development purposes in collaboration with academia and the public and private sectors.

### Research Design

**Figure 1:**
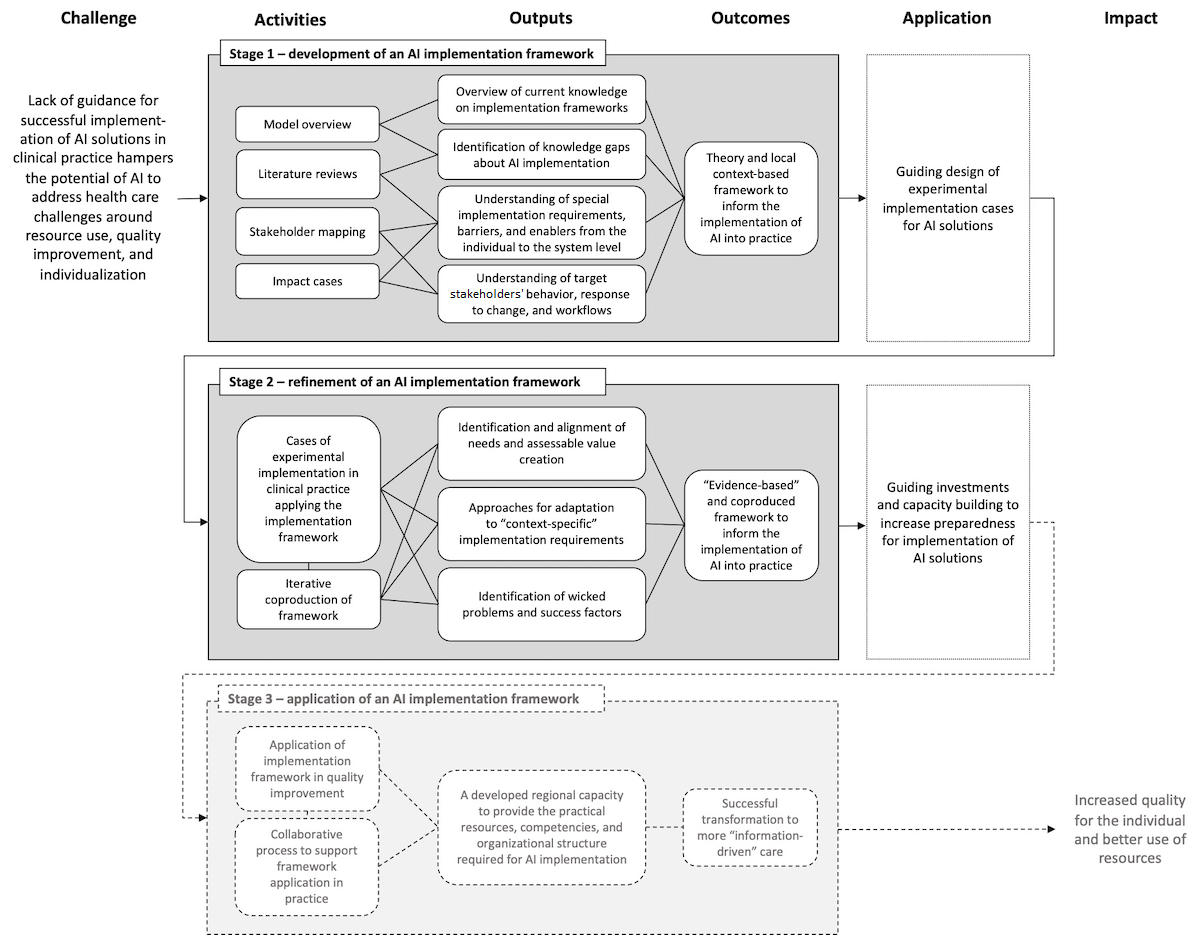
Logic model for the program, including activities identified as necessary to achieve the intended outputs and outcomes in relation to the desired impact and the defined challenge. AI: artificial intelligence.

This overall program can be described using a logic model [34] and will contribute to the development of a methodological framework [35] for planning, facilitating, and evaluating implementation of AI systems in health care and to support capacity building for its use in practice (Figure 1)*.* The logic model is based on common challenges within health care systems regarding achieving and maintaining quality and optimizing the use of available resources, at the same time as the system undergoes a structural transformation with increased digitization and individualization of health care processes, workflow, and organization. A combination of activities has been identified as necessary to achieve the outputs required to accomplish the desired change within the scope of predefined outcome goals.

The logic model has been developed to address issues about implementation through further understanding of the social, cultural, and organizational challenges for implementation and the potential value that can be created from drawing on different perspectives throughout the health care system. In our case, the context for the use of the logic model is AI systems in health care, but it could be equally relevant for studying, planning, facilitating, and evaluating implementation of any other type of technology in health care.

The logic model is divided into three time-separated stages, with a focus on theory-driven and coproduced framework development. The activities are based on both knowledge development, using existing theory and literature reviews, and method development by means of co-design and empirical investigations. The activities involve researchers, health care professionals, and other stakeholders, thus creating a multi-perspective understanding of how the implementation of AI systems should be approached in order to increase likelihood of successful implementation and application in clinical practice.

The framework development spans across the logic model. Workstreams will address the following: (1) identification of evidence to inform the methodological framework (first stage), (2) development of the methodological framework (first stage), and (3) evaluation and refinement of the methodological framework through empirical studies (second stage) [35].

The third stage of the logic model will go on to focus on the practical application of the developed framework in clinical practice. This stage lies further ahead in time and requires work that is more closely integrated with quality-improvement work in clinical practice. It is, therefore, beyond the scope of the first phase of the research program and is not addressed further in this protocol (Figure 1).

### Stage 1: Development of an AI Systems Implementation Framework (Time Frame 2021-2023)

#### Outline

The first stage of this research program focuses on exploratory research with the aim to generate an overview and understanding of the area of interest (ie, “implementation of AI systems in health care”). This will be achieved through the following activities:

1. Development of a preliminary version of a theoretical framework that accounts for different perspectives in implementation in health care in the current literature.
2. Literature reviews of potentially relevant existing theories, models, and frameworks for guiding AI systems implementation in health care and empirically based experiences from implementing AI systems in health care.
3. Mapping of stakeholder perspectives and local awareness and boundaries for AI systems implementation.
4. Theory development based on empirical findings and insights from several initiatives for tentative or pilot implementation of AI systems in health care practice.

#### Model Overview

The first activity is the framing of perspectives on implementation to be incorporated in a theoretical framework based on current literature.

##### Design and Aim

This activity will investigate aspects on implementation and improvement found in models and frameworks in relation to health care [35]. This will give an overview of the different multidisciplinary perspectives on implementation and improvement that need to be considered in order to structure work for successful implementation of new AI systems in health care.

##### Data Collection and Analysis

The investigation will be informed by frameworks describing different perspectives on implementation in health care: service-centric, innovator-centric, and evaluation-centric perspectives. These three perspectives represent the different interests of the major stakeholder groups that we will be engaging (ie, health care professionals and patients, industry partners, and academics). In our aim to co-design a framework that engages all of these groups, we believe it is important to understand the perspectives of individual groups, to shape and understand how the different perspectives come together, and to identify where synergies and tensions are likely to exist.

We have identified three available frameworks that represent these three perspectives, and these will form a basis for initial conceptual development of the framework, which will subsequently be informed by wider literature in each of these fields and empirical data specific to AI implementation. The frameworks we have selected represent these diverse viewpoints; they are leading frameworks in their respective fields and are based on extensive literature review and empirical study. The frameworks are as follows: the Successful Healthcare Improvements From Translating Evidence in Complex Systems (SHIFT-Evidence) framework [29]; the nonadoption, abandonment, scale-up, spread, and sustainability (NASSS) framework [36]; and the process evaluation of complex interventions (PECI) framework [37]. 

The service-centric SHIFT-Evidence framework contains three strategic principles: (1) “act scientifically and pragmatically,” (2) “embrace complexity,” and (3) “engage and empower.” These provide actionable guidance to inform the implementation and improvement process. In comparison to other leading implementation frameworks, SHIFT-Evidence offers a service-centric framework for conceptualizing the work required for successful implementation as comprised of an ongoing and multifaceted process of intervening in complex health care systems [38]. The framework makes clear that innovation implementation alone is unlikely to achieve improvements in care unless the wider interdependent issues are understood and addressed, including how the service is run and the behaviors of staff and patients. The framework emphasizes the real-world and often messy environments of service providers and service users that need to be the foundation of real-world implementation if it is to succeed in achieving improvements. Therefore, this framework is a sensible choice to ensure that a holistic approach is taken to AI systems implementation.

The innovator-centric NASSS framework comprises six domains: (1) the condition or illness, (2) the technology, (3) the value proposition, (4) the adopter system (ie, comprising professional staff, patients, and lay caregivers), (5) the organizations, and (6) the wider institutional and societal context; a seventh domain considers interactions and adaptations over time [36].

The NASSS framework adopts an innovator-centric view of the spread of technology and, therefore, complements the service-centric view of the SHIFT-Evidence framework. In addition, the NASSS framework focuses on the specific challenges related to technological innovations. While this is not specific to AI systems, there is considerable overlap in the influencing factors of general technological interventions (eg, the types of data generated, the knowledge needs to use the technology, the technology supply model, understanding the value proposition for the supply side [ie, developer] and demand side [ie, patient and service], the regulatory environment, and the position of professional bodies). 

The evaluation-centric PECI framework [37] provides actionable guidance to evaluate the implementation process. It does so by examining implementation factors (ie, the process through which interventions are delivered, and what is delivered in practice), mechanisms of impact (ie, the intermediate mechanisms through which intervention activities produce intended or unintended effects), and contextual factors (ie, factors external to the intervention that may influence its implementation, or whether its mechanisms of impact act as intended).

Data on different aspects within these frameworks will be extracted from the original manuscripts for each implementation framework. Assessment will consider both the number of related aspects and the breadth of issues they address. Thereafter, a literature search of implementation frameworks or models for health care will be conducted; identified literature will then be deductively analyzed to extend the understanding of the concepts identified in the initial analysis and to potentially identify, describe, and amend further concepts. This is an iterative process; after deductively synthesizing the new data, they will be discussed with key experts, researchers, and potential framework users (eg, health care and industry partners) for refinements [35].

#### Literature Reviews

The second activity is the completion of two scoping reviews investigating the following: (1) frameworks and models used to guide implementation of AI systems in health care and (2) empirical reports of AI systems implementation.

##### Design and Aim

Two scoping reviews based on the framework by Arksey and O’Malley [39] will be conducted in four stages: identification of relevant literature, selection of studies, charting of data, and synthesizing results. Scoping reviews are well suited to fields that are not yet comprehensively reviewed [39,40]. This methodology allows for summarizing the current state of the art, to map key findings, to identify research gaps, and to make recommendations for future research and practices [40]. By using search terms that capture general aspects of implementation, all three perspectives (ie, service-centric, innovator-centric, and evaluation-centric perspectives) will be covered in the reviews. This will allow for evaluation of how these perspectives have influenced current research on AI implementation in health care and where there is agreement or conflicting knowledge between the three perspectives.

Two reviews will be performed with separate but interrelated aims. The first review will investigate the existence and use of AI-specific implementation frameworks and identify which ones have been used to understand and support AI systems implementation in health care. The second review will investigate the existing empirical research on AI implementation and what lessons can be learned from this research for potential application in the development of an AI-specific framework for implementation in health care. This will allow us to map and synthesize barriers and facilitators for successful implementation into health care practice.

##### Data Collection and Analysis

Electronic databases—Cochrane, EBM Reviews, Embase, MEDLINE, and PsycInfo—will be searched to identify publications that were published in the last 10 years. The reviews will focus on studies published in English and investigating issues concerning AI systems implementation in health care. Two reviewers will independently review the titles and abstracts of the identified papers on the basis of inclusion and exclusion criteria using the Rayyan web platform. Any disagreements will be resolved by involving a third independent reviewer. Following the title and abstract review stage, full texts of identified papers will be obtained for more thorough review. The data will be extracted from the final set of studies using data extraction forms, including bibliographic details of the study, population and age group studied, geographical location, contexts where the studies were carried out, the dimensions of the implementation that were explored, and the specific AI systems of interest. Results will be collated, summarized, and reported deductively using the frameworks (ie, service-centric, innovator-centric, and evaluation-centric) investigated during the first activity. 

#### Stakeholder Mapping

The third activity is the mapping of stakeholders and local awareness and boundaries for AI systems implementation.

##### Design and Aim

This activity has an exploratory qualitative approach [41,42] in order to understand contextual aspects regarding requirements, barriers, and enabling factors for the introduction of AI systems and, thus, their development and plan for implementation. The aim is to inform the planning process for AI systems implementation, to ensure that developed strategies to support suitable implementation approaches are based on stakeholder perspectives, and to avoid potential barriers to AI systems integration in clinical practice. The studies seek to answer the following research questions:

- How can stakeholder perspectives be used to help understand risks and opportunities in relation to AI systems implementation in health care?
- What assumptions are made by different stakeholders about the system and people? What opportunities and risks do the stakeholders perceive?
- How is the potential adoption of AI systems conceptualized by different stakeholders who are responsible for or impacted by AI systems? 

##### Data Collection and Analysis

Key individuals, groups, or both who can affect or are affected by the implementation of AI systems in health care will be identified based on a high-level managerial starting point, and will continue to be identified through a snowball recruitment procedure. Recruitment will begin based on different settings, starting at the regional health care strategic management level and continuing with two frontline health departments, within which AI systems are intended to be developed and implemented. Within these settings, data in relation to implementation of AI systems will be collected from the management level, development level, and clinical practices. Individual interviews will be conducted with stakeholders representing different needs, experiences, interests, mandates, and responsibilities and will continue until the informants do not identify any further types of stakeholder perspectives to be included. This procedure will allow for informant perspectives to be represented, including from health care professionals, managers and quality developers, IT technicians, politicians, and patients, among others.

The interviews will be based on an interview guide structured around the following: (1) the roles and previous experiences the informants have concerning the application of AI systems in practice, (2) the opportunities and problems that need to be solved and considered to create strategies to support suitable introduction of AI systems, (3) beliefs and attitudes concerning the possibilities of using AI systems to support health care improvements, and (4) the obstacles, opportunities, and facilitating factors that need to be considered to enable AI systems to fit into existing processes, methods, and systems.

The analysis will be both inductive and deductive and will be structured by qualitative content analysis [41] and stakeholder mapping approaches [43]. Qualitative content analysis largely focuses on the subject and context, exploring differences between and similarities within codes and categories [41,42]. The analysis method is chosen based on the need to structure and condense various aspects of implementation that are described by the stakeholders based on the material collected. Interview data will be analyzed by (1) identifying stakeholders and their efforts in the ecosystem of services and their interest versus influence, (2) investigating different stakeholders’ perspectives and assumptions regarding AI systems in health care (ie, inductive qualitative content analysis), and (3) investigating how the potential adoption of AI systems is conceptualized by different stakeholders (ie, deductive qualitative content analysis) using the frameworks (ie, service-centric, innovator-centric, and evaluation-centric) investigated during the first activity.

Qualitative content analysis, both with an inductive and deductive approach, consists of several analysis steps [41]. Data analysis will be led by one researcher, but he or she will collaborate with other researchers in order to increase the credibility and trustworthiness of the interpretation [42]. We will seek agreement between the researchers and continuously discuss how well the codes, categories, and themes represent the data. The researchers will work both individually and together during the analysis process until consensus is reached.

#### Impact Cases

The fourth activity is developing theory based on experiences from several initiatives for tentative or pilot implementations of AI systems in practice.

##### Design and Aim

This activity takes an exploratory qualitative approach to understand the different competencies, knowledge, perspectives, and logics that are represented by the different included or affected stakeholders, their professional roles, and their organizations in relation to the introduction of AI systems in clinical practice. The study will provide information on the preimplementation social, professional, and organizational context and structures that exist in the different arenas of the health system and will provide a base analysis of current practical, professional, organizational, and clinical structures and arrangements. The aim is to inform the planning process of AI systems implementation and to ensure that developed strategies to support suitable implementation approaches are based on the different social, cultural, practical, and organizational boundaries that will affect the integration of AI systems in clinical practice, as well as on how those social structures change through the process of AI systems implementation. The cases will provide a foundation for answering the following research questions:

- What are the special implementation requirements, barriers, and enablers specific to AI systems in health care?
- What are the target stakeholders’ behaviors, responses to change, and new workflows as a result of introducing AI systems in health care?
- How do social structures and roles change through the process of AI systems implementation, particularly in relation to implementation requirements, barriers, and enablers?

##### Data Collection and Analysis

In this phase, a wide variety of data will be included in a qualitative thematic analysis [44] in the form of stakeholder interviews and meeting notes, as well as observations, plans, and reports from cases. Data will be collected on a set of concrete and specific cases of potential, tentative, or ongoing implementation of AI systems. Included cases will consist of the research, development, and innovation projects carried out during the project period within the region’s and university’s research and development activities. The cases will mainly be identified through the steering group for the research center CAISR Health, which includes leadership representatives from the university, the region, and business partners.

The purposively selected projects will allow for the collection of a diverse body of data on a wide range of practical aspects of AI system implementation, including data collection in formative studies (ie, through observations, interviews, and surveys), design and development studies (ie, interviews, focus groups, workshops, and usability and feasibility evaluations), and intervention and implementation studies (ie, interviews, quantitative process and effect evaluations, and health economy assessments). The data analysis method will be chosen based on the need to structure and condense the various aspects of implementation that can be drawn from a variety of data sources from different types of cases. In combination with the analysis from the third activity, this analysis will help identify the different types of barriers and enablers for AI system implementation as well as the social and organizational processes that maintain and create them.

### Stage 2: Refinement of an AI Systems Implementation Framework (Time Frame 2023-2026)

#### Outline

The second stage of this research program focuses on implementation research with the aim of further identifying and aligning needs with assessable value creation in the implementation of AI systems. The objective is to develop a better understanding of the approaches that can be taken to adapt implementation strategies and AI systems to context-specific implementation requirements. Developing such approaches depends on exploration of the nature of wicked problems and success factors that can hinder or facilitate successful implementation in practice. This will be achieved through the development and execution of several implementation projects, together with partners from public and private health care and companies, and through an iterative process for coproduction of a refined framework. 

#### Implementation Studies

##### Design and Aim

Each implementation study will be initiated and designed based on conversations and workshops with representatives from public and private care providers, companies that develop or apply AI technology and service development, and researchers. A network of such actors has been established through the formation of CAISR Health and will be expanded and developed through, among other approaches, the built-up infrastructure that is linked to the national innovation environment for information-driven care, Leap for Life, RHIP, CIDD, and HDC. The design and aim of each study will be adapted based on each study’s specific objectives and context. For each study, the study design will be based on the structure and requirements of the implementation framework developed in Stage 1. The cases for the studies will be selected and used to explore and develop the implementation framework, with each case chosen to enable in-depth examination of particular aspects of the implementation framework and the broader sociotechnical system around AI technology, particularly with regard to the support and improvement of clinical workflows, processes, and systems.

##### Data Collection and Analysis

For each implementation study, data will be collected through qualitative and quantitative methods (project documentation, interviews, evaluations, etc) to examine the extent to which the implementation was carried out according to the plan developed based on the implementation framework. Data collection will focus on capturing aspects such as what deviations were made and why they were made; which aspects of the implementation worked well and what problems arose; how governance, management, and assurance processes were established around, and as part of, these new AI systems, and the impacts these processes had on AI system implementation and use; and whether implementation achieved the desired change, whether it contributed to an increased value, and, if so, for whom. Each implementation study will function as a separate project with its own project management and goals.

The analysis of the continuously collected data will be based on the grounded theory methodology [45]. In this methodology, the researcher explores how individuals construct meaning and actions, and the researcher aims to examine the context, existing structures, hierarchies of power, networks, and relationships in which the actions and processes take place. Analysis will be conducted by iteratively coding and categorizing the qualitative data that are collected, in order to develop higher-order concepts and explanations of the processes and challenges of AI implementation. The methodology is suitable because it enables exploration and understanding of various actions and processes involved in AI implementation. The aim is to develop a theoretical model that can inform policy and practice with regard to AI implementation. We will follow the quality criteria for constructivist grounded theory (ie, credibility, resonance, originality, and usefulness) [45].

By running all implementation studies as part of a larger program collaboration over several years, the joint documentation around each implementation study will contribute knowledge from many different perspectives and contexts. This will provide a foundation for continuous refinement and development of the framework for AI systems implementation developed in Stage 1. 

#### Iterative Coproduction of Framework

##### Design and Aim

The constructivist grounded theory approach will be used [45] to develop, revise, and refine the previously generated framework (Stage 1) through the analysis of new empirical data and experiences of stakeholders who have worked practically with the framework in the implementation of AI systems in practice. This will be done to examine practices, roles, role relationships, and the situated and material organizational aspects of using the framework and implementing AI. The main objective is to iteratively refine the framework developed in Stage 1 in coproduction with stakeholders, in order to produce an evidence-based and coproduced framework that accounts for appropriate strategies that will enable the production of relevant value in practice.

##### Data Collection and Analysis

The grounded theory approach will guide iterative data collection and analysis of a range of documentation from implementation studies, such as project plans, interviews, observations, surveys, workshops, and meeting notes. This process will inform the interpretation and refinement of the framework. Grounded theory has been chosen, as it focuses on human experiences and actions in social contexts [45]; the theory is appropriate, since the phenomenon of implementing AI systems in practice is a complex area that involves many different people, actors, social structures, and processes. Members of a core team will be identified from the research group and from the different implementation studies to work together throughout the coproduction process in order to leverage different perspectives, experiences, and insights. The coproduction process involving the core team will be interactive and iterative, and will involve various management functions and stakeholders with experiences of the implementation of AI systems in health care. Particular focus will be placed on the interplay between theory and empiricism, in order to explain novel empirical data and how this data can modify and challenge the existing concepts and theoretical constructions in previous iterations and versions of the framework generated in Stage 1.

### Ethics and Dissemination

Ethical approval will be applied for regarding all empirical parts of the program. Site-specific approvals will be obtained for each site prior to commencing study activities. The study conforms to the principles outlined in the Declaration of Helsinki [46] and will fulfill the following requirements for research: information, consent, confidentiality, and safety of the participants guided by the ethical principles of autonomy, beneficence, nonmaleficence, and justice. All participants will receive written and verbal information about studies in which they are directly or indirectly involved. Participants will also be given information about the voluntary nature of the studies, confidentiality, and the ability to withdraw their consent at any time without having to justify why. All personal data will be registered according to the General Data Protection Regulation (GDPR2016/679), and the data will be stored in accordance with the Archive Act in Sweden (SFS1990:782).

The results of this program will be communicated to the included participants and partners, and key findings will be fed back to sites to enable refinement of strategies for implementation of AI systems in health care. The results will be disseminated via publications in peer-reviewed journals and presentations at national and international conferences.

## Results

**Figure 2:**
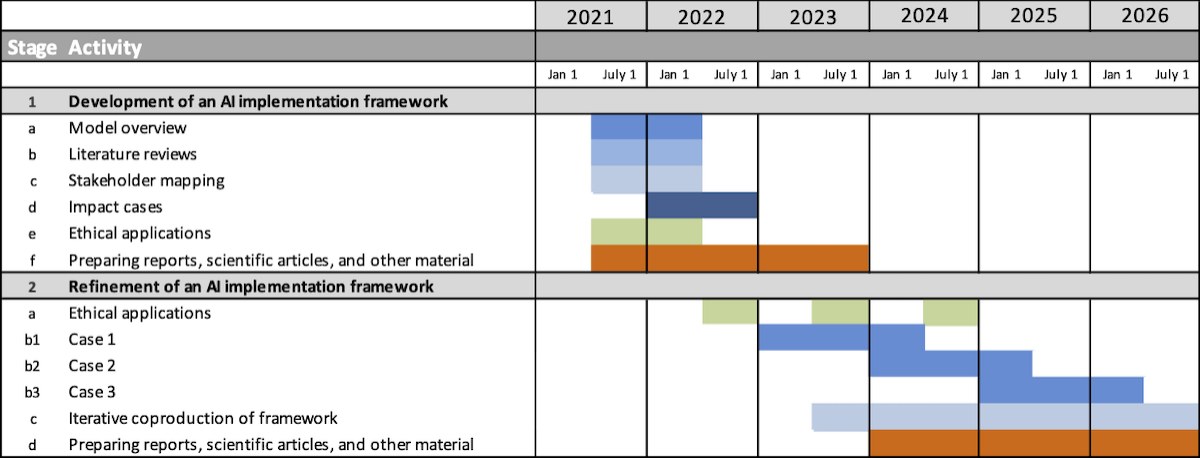
Project timeline. AI: artificial intelligence.

The purpose of Stages 1 and 2 is to develop a framework that specifically describes how implementation of AI systems in health care should be approached to increase the likelihood of successful implementation and use in routine health care practice. In Stage 1, starting in July 2021, the first version of the implementation framework started to be developed based on the initial investigation of current knowledge on implementation and improvement frameworks and models in health care (Figure 2). The evolution of the framework will thereafter be guided by findings from the literature reviews, stakeholder interviews, and impact cases to supplement it with current scientific evidence and relevant contextual conditions. In Stage 2, starting in July 2023, several implementation cases will be implemented with different sizes, time frames, and scopes of ambition (Figure 2). These implementation studies will be the means for developing the framework for AI systems implementation, which is, therefore, seen as the main product under Stage 2. The framework will be under continuous development during Stage 2 and will be reported in scientific articles and other formats for more practical application. The understanding and knowledge from Stage 2 can thereafter be used as the foundation for investments in a developed regional capacity to increase the practical resources, competencies, and organizational structure required for AI systems implementation, in collaboration with academia, industry partners, and health care. The planning for the integration of the outcomes from this research program will take place in parallel with the work on Stages 1 and 2 to allow for continuation and translation of the research to strategic investments in quality improvement in clinical practice (Stage 3 in Figure 1). This work will involve representatives from the university, the political and operational management teams of the regional and municipal health care systems, strategic partners from the business community, and representatives from various patient-specific interest groups.

## Discussion

There is an immediate need to understand the health care service pathways and processes for AI systems to ensure that their impact in widespread practice fulfills the promise of transforming health care data into meaningful and actionable insights that support decision-making, optimize care processes, and provide high-quality patient care. Knowledge generated from implementation science and improvement science could be useful for understanding and developing methods, models, and frameworks needed to promote the uptake of research findings on AI systems into health care practices. The knowledge and expertise in implementation research in health care, in general, is advancing, but this is mainly held by research experts in the field. This knowledge is not widely accessible or used by people who could benefit from it, meaning that implementation efforts are not as successful as they could be, and common mistakes are repeated. There is a need to advance theory and empirical evidence regarding implementation requirements of AI systems in health care, and an opportunity to bring together insights from research on the development, introduction, and evaluation of AI systems and existing knowledge from implementation research literature. Therefore, with this research program, we intend to build an understanding, using both theoretical and empirical approaches, of how implementation of AI systems should be approached in order to increase the likelihood of successful and widespread application in clinical practice. 

## Abbreviations

AI: artificial intelligence
CAISR: Center for Applied Intelligent Systems Research
CIDD: Center for Information-Driven Care
HDC: Health Data Center
HU: Halmstad University
IT: information technology
NASSS: nonadoption, abandonment, scale-up, spread, and sustainability
PECI: process evaluation of complex interventions
RH: Region Halland
RHIP: Regional Healthcare Information Platform
SHIFT-Evidence: Successful Healthcare Improvements From Translating Evidence in Complex Systems
